# Clinical Integration of NIR-II Fluorescence Imaging for Cancer Surgery: A Translational Evaluation of Preclinical and Intraoperative Systems

**DOI:** 10.3390/cancers17162676

**Published:** 2025-08-17

**Authors:** Ritesh K. Isuri, Justin Williams, David Rioux, Paul Dorval, Wendy Chung, Pierre-Alix Dancer, Edward J. Delikatny

**Affiliations:** 1 Department of Radiology, Perelman School of Medicine, University of Pennsylvania, Philadelphia, PA 19104, USA; 2 Department of Chemistry, School of Arts and Sciences, University of Pennsylvania, Philadelphia, PA 19104, USA; 3 Department of Physics, College of Liberal Arts and Sciences, Villanova University, Villanova, PA 19085, USA; 4 Photon etc., 5795 Ave de Gaspé, #222, Montréal, QC H2S 2X3, Canada; 5 Kaer Labs, 1 Rue Julien Videment, 44200 Nantes, France

**Keywords:** NIR-II fluorescence imaging, fluorescence-guided surgery, intraoperative imaging systems, LightIR, IR VIVO, cancer surgery

## Abstract

**Background/Objectives**: Back table fluorescence imaging performed on freshly excised tissue specimens represents a critical step in fluorescence-guided surgery, enabling rapid assessment of tumor margins before final pathology. While most preclinical NIR-II imaging platforms, such as the IR VIVO (Photon, etc.), offer high-resolution and depth-sensitive imaging under controlled, enclosed conditions, they are not designed for intraoperative or point-of-care use. This study compares the IR VIVO with the LightIR system, a more compact and clinically adaptable imaging platform using the same Alizé 1.7 InGaAs detector, to evaluate whether the LightIR can offer comparable performance for back table NIR-II imaging under ambient light. **Methods**: Standardized QUEL phantoms containing indocyanine green (ICG) and custom agar-based tissue-mimicking phantoms loaded with IR-1048 were imaged on both systems. Imaging sensitivity, spatial resolution, and depth penetration were quantitatively assessed. LightIR was operated in pulse-mode under ambient lighting, mimicking back table or intraoperative use, while IR VIVO was operated in a fully enclosed configuration. **Results**: The IR VIVO system achieved high spatial resolution (~125 µm) and detected ICG concentrations as low as 30 nM in NIR-I and 300 nM in NIR-II. The LightIR system, though requiring longer exposure times, successfully resolved features down to ~250 µm and detected ICG to depths ≥4 mm. Importantly, the LightIR maintained robust NIR-II contrast under ambient lighting, aided by real-time background subtraction, and enabled clear visualization of subsurface IR-1048 targets in unshielded phantom setups, conditions relevant to back table workflows. **Conclusions**: LightIR offers performance comparable to the IR VIVO in terms of depth penetration and spatial resolution, while also enabling open-field NIR-II imaging without the need for a blackout enclosure. These features position the LightIR as a practical alternative for rapid, high-contrast fluorescence assessment during back table imaging. The availability of such clinical-grade systems may catalyze the development of new NIR-II fluorophores tailored for real-time surgical applications.

## Simple Summary

Fluorescence imaging is increasingly being used during cancer surgery to help surgeons delineate tumors more clearly, but most current systems work in the first near-infrared (NIR-I) range, which limits how deeply light can penetrate tissue. This can make it hard to detect tumors that are just below the surface. In this study, we compared two imaging systems that operate in the second near-infrared (NIR-II) range, which allows for deeper tissue imaging with less background noise. One system is used in research labs, while the other is designed for real-time use in surgery without needing to darken the room. We tested both systems using special dye-filled models that mimic human tissue. Our findings demonstrate that the LightIR system enables NIR-II fluorescence imaging under ambient light, supporting its practical use in real-time intraoperative settings.

## 1. Introduction

In recent years, surgical oncology has witnessed a paradigm shift with the integration of advanced imaging modalities designed to enhance intraoperative guidance. Precise delineation of tumor margins is paramount for effective cancer resection, as residual malignant tissue can lead to recurrence and poor patient outcomes [1,2]. Fluorescence-guided surgery (FGS) has emerged as a transformative approach in this context, enabling real-time visualization of anatomical structures and tumor boundaries, thereby facilitating higher precision in excision procedures [1,3,4,5,6,7,8,9,10,11,12,13,14,15,16,17].

Despite the progress made with earlier near-infrared (NIR) imaging systems, several limitations intrinsic to conventional imaging agents hinder optimal surgical outcomes [18]. Traditional methods have relied heavily on the usage of dyes with fluorescence emissions primarily in the first near-infrared window (NIR-I, 700–950 nm), such as Indocyanine Green (ICG). ICG is widely employed for mapping sentinel lymph nodes, angiography, and intraoperative tumor detection, all of which are due to its established clinical safety profile and ease of use [2,19]. However, the use of NIR-I dyes is subject to fundamental drawbacks, including limited tissue penetration, high autofluorescence, and low signal-to-background contrast [2,20]. These constraints reduce the fidelity of intraoperative imaging, resulting in suboptimal demarcation of tumor margins [18]. Furthermore, the photophysical properties of ICG, such as moderate fluorescence quantum yields and strong plasma protein binding, contribute to its limited contrast and rapid clearance, complicating image interpretation during surgery [2,21].

Emerging evidence has highlighted the potential of the second near-infrared window (NIR-II, 1000–1700 nm) to overcome many of these obstacles. NIR-II imaging offers deeper tissue penetration, improved spatial resolution, and notably reduced background autofluorescence [22,23,24,25]. These advantages are primarily attributed to the reduced scattering and lower absorption coefficients present in tissue at longer wavelengths [23,26]. A number of studies have demonstrated that the use of NIR-II fluorophores can enhance tumor-to-background ratios significantly, facilitating more accurate discrimination between malignant and healthy tissue [27,28,29]. Beyond improved imaging contrast, the enhanced tissue penetration of NIR-II light facilitates visualization of structures located several millimeters below the tissue surface—a critical requirement in surgeries involving deep-seated tumors [23,30,31]. Such improved imaging performance has been corroborated in various preclinical models, where NIR-II imaging has successfully delineated vascular networks and tumor margins with unprecedented clarity [22,32,33,34,35].

Several preclinical studies have shown promise in applying NIR-II imaging to various cancer models. For example, research on ovarian and breast cancer models has demonstrated that NIR-II nanoprobes can reliably delineate tumor margins, facilitating both surgical planning and intraoperative guidance [36,37]. Likewise, the application of NIR-II imaging in vascular and lymphatic mapping has proven invaluable in identifying critical structures that must be preserved during surgery [22,32]. These studies are pivotal in establishing the rationale for the clinical translation of NIR-II imaging systems, which must be both sensitive enough to detect minute tumor deposits and robust enough to function under diverse surgical conditions [3,38].

However, current NIR-II imaging technology has limitations primarily attributed to challenges related to fluorophores, costs, and biocompatibility. One significant disadvantage is the limited pool of effective NIR-II fluorophores, which often suffer from low quantum yields and undesirable pharmacokinetics, impacting their utility in clinical settings [27,39]. The lack of biocompatible and highly efficient fluorophores furthers this issue, as many inorganic options raise safety concerns due to potential accumulation and immunogenic responses in vivo [27]. Furthermore, the photostability and brightness of existing organic NIR-II probes are suboptimal, leading to diminished imaging quality and limited operational usage over time [40,41]. Cost is another hurdle; advanced equipment required for NIR-II imaging, such as specialized cameras and detectors, can be prohibitively expensive, thus limiting accessibility and widespread clinical application [42]. Finally, while potential alternatives such as peptides are being explored to counter the long blood clearance time associated with antibody-based probes, advancements in this area remain nascent [43]. These limitations collectively impede the broader implementation of NIR-II imaging technologies in medical and research environments.

As the field moves forward, the synergistic progression of fluorophore chemistry, imaging hardware, and computational analysis promises to revolutionize intraoperative imaging. The ongoing development of targeted NIR-II probes, combined with refined imaging systems, lays the groundwork for a future where image-guided surgery is not only more accurate but also more accessible across different surgical specialties [22,28]. The success of such technologies will ultimately be measured by their ability to improve patient outcomes through enhanced precision in tumor resections, marking a significant leap in the fight against cancer [36].

While advances in NIR-II fluorophore development have been impressive, the translation of these benefits to clinical practice necessitates the parallel evolution of imaging instrumentation. Currently, many imaging systems are either optimized for preclinical research or tailored to the clinical environment, but few systems exist that effectively bridge both domains [3,38,44,45]. The fundamental challenge lies in designing imaging platforms that can harness the full potential of NIR-II probes, ensuring high sensitivity and resolution while maintaining user-friendly operation in the operating theatre [3,46]. For instance, systems based on InGaAs detectors have been crucial in detecting the longer wavelengths of NIR-II fluorescence; however, factors such as cost, signal stability, and integration with other surgical equipment remain key hurdles [47].

The IR VIVO is an NIR preclinical imaging system that has been optimized for the detection of NIR-II emission, enabling high-speed, high-resolution imaging that is indispensable in preclinical studies [22,25,30,48,49,50,51,52,53,54,55]. However, the IR VIVO is a relatively bulky system that, once installed, is not easily portable. While well-suited for preclinical applications, its design limits its use in clinical settings. In this paper, we present a comparison between the IR VIVO system and the Light-IR, a new intraoperative NIR-II imaging system that aims to meet the demands of the clinical environment by integrating NIR-II detection with real-time surgical navigation and decision-making processes. Both the IR VIVO and the LightIR utilize the Alize 1.7 InGaAs camera; however, the LightIR is designed to be more compact and portable, making it better suited for clinical use. These two systems represent critical steps towards overcoming the dichotomy between preclinical and clinical imaging infrastructures, ensuring that advances in fluorescence probe design translate effectively into improved patient outcomes, underscoring the translational potential of NIR-II technologies for enhancing surgical outcomes.

## 2. Materials and Methods

### 2.1. Imaging Systems

#### 2.1.1. Preclinical Imaging—IR VIVO (Photon, etc.)

Preclinical fluorescence imaging was performed using the IR VIVO system (Photon etc., Montréal, QC, Canada), a small-animal imaging platform optimized for NIR-II (short-wave infrared, SWIR) detection. The system contains a thermoelectrically cooled 640 × 512 pixel InGaAs detector coupled to a large aperture detection lens (optimized for NIR-II imaging, delivering ≤100 µm spatial resolution across a field of view sufficient for whole-body mouse imaging. Excitation is provided by interchangeable continuous-wave laser diodes at 760, 808, 890, and 940 nm with emission collected via a motorized filter wheel equipped with long-pass filters (NIR-I, LP1000, LP1250). For NIR-I imaging, the system uses a 975 nm short pass filter in combination with a laser-dependent long pass filter, an 800 nm filter for 760 nm excitation, an 850 nm filter for 808 nm excitation, a 925 nm filter for 890 nm excitation, and a 975 nm filter for 940 nm excitation. The power density of the IR VIVO for different lasers and imaging platform height combinations is listed in Table A1. The system supports rapid switching between excitation/emission settings, real-time spectral unmixing, radiant efficiency calibration, and signal-to-background quantification. An integrated anesthesia manifold and heated stage allow for longitudinal animal studies under physiologically controlled conditions.

#### 2.1.2. Intraoperative Imaging—LightIR (Kaer Labs)

Clinical-style intraoperative imaging was conducted using the LightIR platform (Kaer Labs, Nantes, France), designed for real-time NIR-II imaging during surgical procedures. The system integrates a scientific-grade Alizé 1.7 InGaAs detector stabilized to −60 °C to suppress thermal noise. Dual high-power CW lasers at 808 nm and 980 nm provide uniform illumination across a 7 × 7 cm field of view, with lateral resolution of 140 µm in standard configuration (upgradable to 100 µm over 5 × 5 cm). A 1050 nm long-pass emission filter is built into the system, with interchangeable filters (LP1100, LP1200, LP1300) for tunable spectral sensitivity. The modular optical head mounts to a surgical cart or articulated arm for hands-free alignment. Real-time video acquisition of up to 30 fps (110 fps in high-gain mode) supports dynamic feedback during fluorescence-guided surgery.

### 2.2. Phantom Fabrication

#### 2.2.1. QUEL Imaging Phantoms

Standardized optical phantoms were obtained from QUEL Imaging (Hartford, VT, USA, www.quelimaging.com), including concentration, depth, and resolution targets. The concentration phantom consisted of nine dilutions of indocyanine green (ICG) from 1000 nM to 0 nM, with one well containing a quantum dot (QD) positive control. The depth phantom contained ICG at 1000 nM, embedded at depths from 0 to 6 mm. The resolution phantom incorporated a USAF 1951 to assess spatial resolution under different imaging conditions. A vessel-mimicking phantom was also obtained from QUEL using one channel filled with 1000 nM ICG and a second channel with blank buffer as a control. This setup was used to simulate anatomical structures and evaluate the imaging system’s ability to resolve vessel-like features under NIR-II conditions.

#### 2.2.2. IR-1048 Depth Phantoms

Custom phantoms for IR-1048 imaging were prepared using agar gel (1.5% *w*/*v*) doped with 1% (*v*/*v*) Intralipid and 0.01% (*v*/*v*) India ink to simulate the scattering and absorption properties of biological tissue. IR-1048 (Sigma-Aldrich, St. Louis, MO, USA) was dissolved in DMSO to a final concentration of 10 µM, and a 5 × 5 mm^2^ sponge was soaked in this solution to localize the dye. The dye-loaded sponge was then embedded at defined depths (0–5 mm) within the agar matrix prior to gelation, to enable evaluation of subsurface NIR-II fluorescence signal attenuation under physiologically relevant optical conditions.

### 2.3. Imaging and Analysis Protocols

#### 2.3.1. Sensitivity and Depth Imaging with ICG

To evaluate the sensitivity and depth-resolved imaging performance of each system, standardized QUEL concentration and depth phantoms containing ICG were imaged across a range of conditions. The phantoms included different ICG concentrations (0–1000 nM) for sensitivity testing and wells with ICG embedded at depths from 0 to 6 mm to assess tissue penetration. All experiments were conducted in triplicate, and results are reported as the mean ± SD.

On the IR VIVO preclinical system, imaging was conducted using excitation wavelengths of 760, 808, 890, and 940 nm. Emission was collected using either NIR-I filters or long-pass filters (LP1000 and LP1250) to isolate NIR-II signals. Exposure times ranged from 6.25 ms to 1000 ms. Signal-to-background ratios (SBRs) were computed using Photon etc’s proprietary analysis software PHySpec (v2.29.2), by comparing the average fluorescence intensity within fluorophore-containing wells to adjacent background regions.

For the LightIR intraoperative system, imaging was performed with 808 nm excitation using the integrated 1050 nm long-pass filter. Exposure times ranged from 2 ms to 1000 ms. Image analysis was performed using ImageJ (v1.54p), where regions of interest (ROIs) were manually drawn around signal and background areas to compute SBRs. This dual-platform analysis enabled a quantitative comparison of imaging sensitivity and depth performance between preclinical and clinically integrated NIR-II systems.

#### 2.3.2. Resolution Testing

Spatial resolution was assessed using a Quel USAF 1951 resolution target phantom. Imaging was performed at a fixed exposure time of 850 ms on both the IR VIVO and LightIR platforms. All experiments were conducted in triplicate, and results are reported as the mean ± SD.

For the IR VIVO preclinical system, resolution testing was conducted across three magnification configurations: Mouse Macro (75 × 60 mm), Macro View (50 × 40 mm), and Super Macro (44 × 35 mm). Each configuration was evaluated under both NIR-I and NIR-II emission filters using 808 nm excitation. Line profiles were extracted from the images using the Photon etc. PhySpec software, and the highest resolvable element was determined by the presence of well-defined peak-trough oscillations in the intensity profile across bar patterns.

For the LightIR intraoperative system, resolution imaging was performed using the default surgical configuration with 808 nm excitation and the built-in 1050 nm long-pass emission filter. The phantom was positioned under the optical head in free-space mode to mimic clinical use conditions. Images were analyzed using ImageJ software to extract horizontal line profiles across the bar targets. The highest resolvable group and element were identified based on distinguishable intensity modulations, enabling spatial resolution estimates in microns (µm).

#### 2.3.3. Depth Imaging and Surgical Guidance with IR-1048

IR-1048-loaded phantoms were imaged under LP1050 long-pass filters using the LightIR system. Quantitative depth-dependent fluorescence intensity was recorded from 0 to 5 mm. For surgical guidance simulation, embedded dye targets at 3 mm and 5 mm depths were visualized and resected under real-time fluorescence feedback, with imaging frames recorded throughout the excision procedure. All experiments were conducted in triplicate, and results are reported as the mean ± SD.

All images were analyzed using Photon etc’s software suite, PHySpec and ImageJ, with subsequent data plotted in MATLAB (v23.2.0.2725701) and GraphPad Prism (v8.0.1) for quantitative interpretation.

After the manuscript was written, Grammarly’s generative AI features were used to suggest sentence rewording and improvements to clarity and flow. These suggestions were reviewed and edited by the author. No AI tools were used to generate original scientific content, data, or interpretations.

## 3. Results

**Figure 1:**
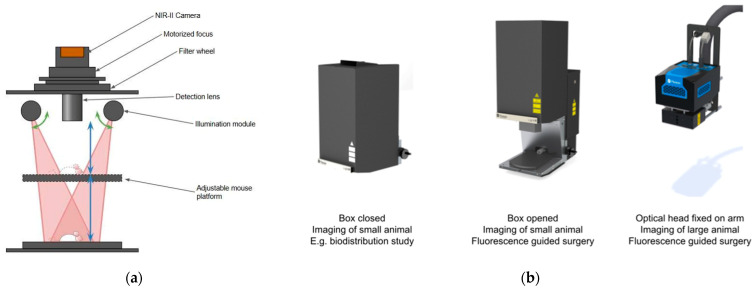
Schematic of the IR VIVO and LightIR systems. (**a**) Cut-away schematic of the IR VIVO platform. A cooled NIR-II camera equipped with a motorized focus unit and integrated filter wheel collects emission light through an interchangeable detection lens. Two angled illumination modules deliver uniform laser excitation (red cones) onto the specimen plane, while an adjustable, height-controlled stage holds the mouse or surgical field at the focal plane; (**b**) Modular configurations demonstrating the versatility of the LightIR system. From left to right, (i) fully enclosed “closed-box” mode for whole-body biodistribution imaging in small animals; (ii) open-box mode, in which the front cover retracts to allow direct instrument access for fluorescence-guided surgery in rodents; and (iii) optical head mounted on an articulated surgical arm for fluorescence-guided procedures in larger animal models or clinical operating theatres.

The optical and mechanical configurations of the IR VIVO and LightIR systems were leveraged to accommodate both preclinical phantom testing and simulated intraoperative procedures (Figure 1). The IR VIVO system was used in its enclosed mode for imaging, with the platform height adjusted to maintain the focal plane for different phantom formats. For LightIR, experiments were conducted using the free-space imaging configuration with the optical head mounted on an adjustable lever. This setup allowed precise adjustment of the working distance between the camera and the surgical field, enabling hands-free alignment during simulated fluorescence-guided surgery tasks.

**Figure 2:**
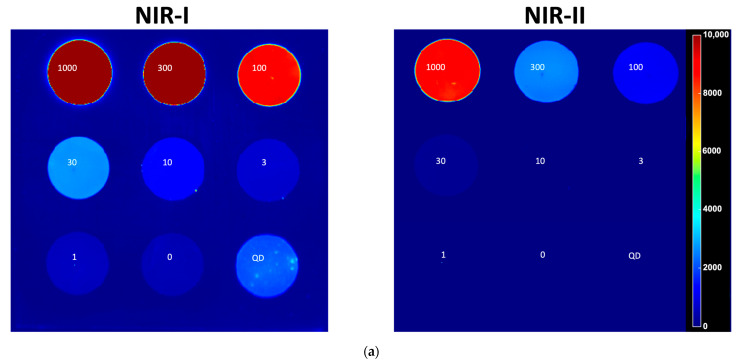

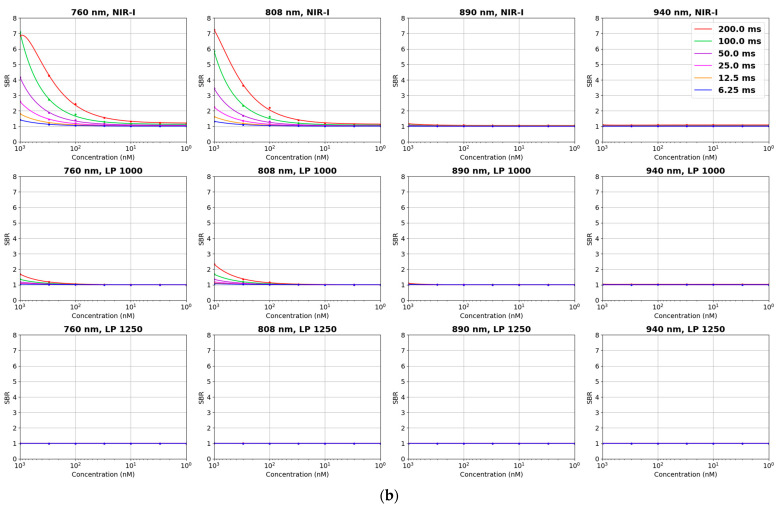
Concentration sensitivity of the IR VIVO for ICG in the NIR-I and NIR-II windows. (**a**) QUEL 3 × 3 concentration phantom (ICG = 1000, 300, 100, 30, 10, 3, 1, 0 nM + QD control) imaged with a standard NIR-I emission filter (Left). The same phantom imaged through a 1000 nm long pass (LP1000) filter to isolate ICG’s NIR-II tail emission (Right). (Pseudo color scale, 0–1 × 10^4^ counts); (**b**) Signal to background ratio (SBR) as a function of concentration for four excitation wavelengths (760, 808, 890, 940 nm; columns) and three emission windows-NIR-I, LP1000, and a 1250 nm long pass (LP1250) filter (rows). Each curve corresponds to one of six integration times (6.25, 12.5, 25, 50, 100, 200 ms; legend).

The sensitivity of the IR VIVO imaging system for detecting ICG was evaluated across a range of excitation wavelengths using a concentration phantom containing 0–1000 nM ICG. In the NIR-I band-pass channel (Figure 2a left), with an exposure time of 200 ms, we obtain the highest signal-to-background ratio (SBR = 7.24) at 1000 nM under 808 nm excitation, with a detectable signal (SBR ≥ 1.41) down to approximately 30 nM. Conversely, at 890 nm and 940 nm, where ICG absorption is minimal, the SBR did not exceed 1.17. When imaging with the 760 or 808 nm laser and NIR-I filter, the detector saturates at exposure times beyond 50 ms, which is why we do not observe any difference across different depths (Figure 2b).

**Figure A1:**
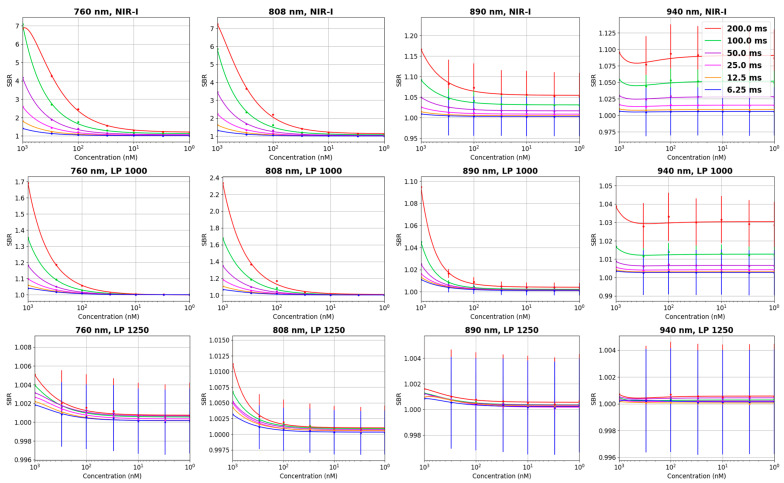
Concentration sensitivity of the IR-VIVO for ICG in the NIR-I and NIR-II windows. Optimal SBRs are shown for each exposure time (6.25–200 ms) across four excitation wavelengths (760, 808, 890, 940 nm; columns) and three emission filters (NIR-I, LP1000, LP1250; rows), illustrating how performance varies with spectral configuration and integration duration.

When emission under 808 nm excitation was captured through a 1000 nm long-pass filter, the ICG tail fluorescence remained measurable only at the two highest concentrations (Figure 2a Right, Appendix A.1, Figure A1) with an exposure time of 200 ms. The maximal SBR dropped to approximately 2.34 at 1000 nM, and the lowest measurable concentration was 300 nM with an SBR of 1.37. A further redshift to the 1250 nm long-pass filter suppressed ICG emission entirely, resulting in SBR values clustering around 1 across all concentrations and exposure times.

**Figure 3:**
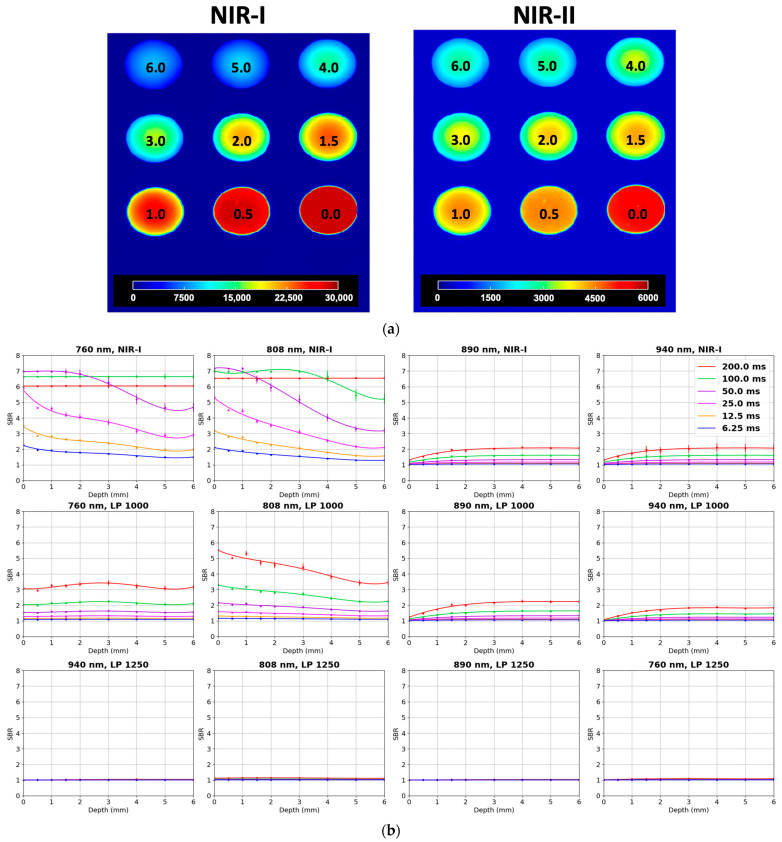
Depth sensitivity of the IR VIVO for ICG in the NIR-I and NIR-II windows. (**a**) QUEL depth phantom containing 1 µM ICG at depths of 0–6 mm imaged in the NIR-I window (Left) (color bar: 0–3 × 10^4^ counts). Identical phantom imaged through the LP1000 filter (Right) (color bar: 0–6 × 10^3^ counts); (**b**) SBR versus tissue depth for four excitation wavelengths (columns) collected with NIR-I (Left), LP1000 (middle), and LP1250 (bottom) emission optics. Each curve corresponds to one of six integration times (6.25, 12.5, 25, 50, 100, 200 ms; legend).

**Figure A2:**
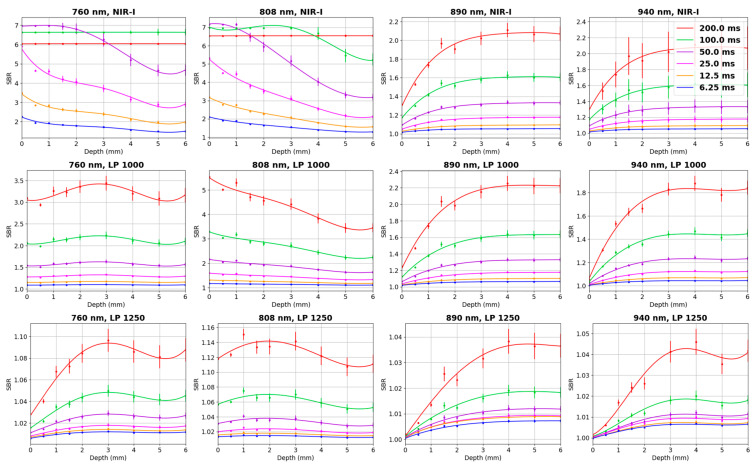
Depth sensitivity of the IR VIVO for ICG in the NIR-I and NIR-II windows. SBR values are plotted for four excitation wavelengths (760, 808, 890, 940 nm; columns) and three emission filters (NIR-I, LP1000, LP1250; rows), with each curve representing one of six integration times (6.25–200 ms). Optimal SBRs for each exposure setting are indicated, demonstrating how depth sensitivity varies with spectral configuration and acquisition duration.

In an experiment using the QUEL depth phantom loaded with 1 µM ICG, we compared the 808 nm excitation collected through a standard NIR-I band-pass filter with a 1000 nm long-pass filter that isolates the weak NIR-II tail emission of the fluorophore (Figure 3a left). In the NIR-I channel, the mean SBR started at 5.31 at the surface (0 mm) and decreased quasi-exponentially to 2.11 at 6 mm (Figure 3b) with an exposure time of 25 ms. In contrast, in order to get a similar SBR under the long-pass filter (LP1000), a longer exposure time of 200 ms was required (5.5 at 0 mm, 3.5 at 6 mm) (Figure 3b, Appendix A.2, Figure A2). Detector saturation at 808 nm excitation occurs as described above. (Figure 3b).

**Figure 4:**
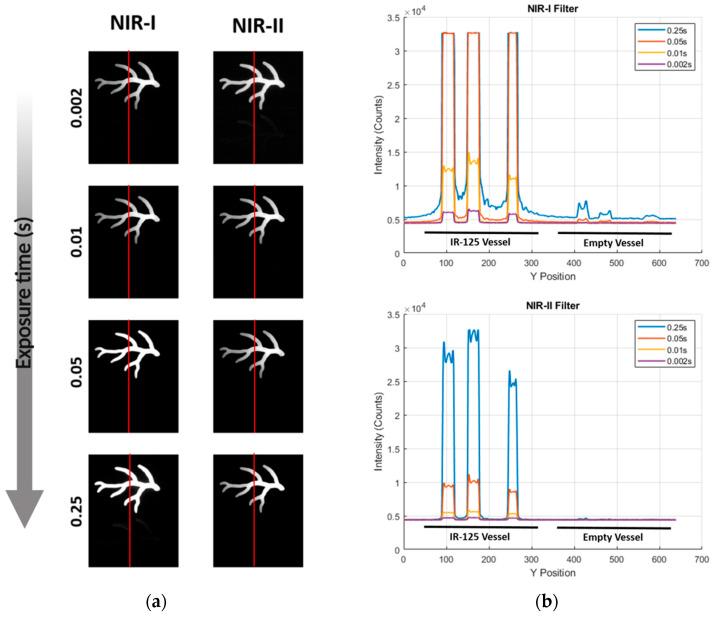
Vessel contrast for ICG in NIR-I versus NIR-II detection. (**a**) Representative images of a vessel phantom containing ICG-filled channels (top) and an empty channel (bottom) acquired at four exposure times (0.002, 0.01, 0.05, 0.25 s; top → bottom) with NIR-I (left) and NIR-II LP1000 (right) emission filters. Red line indicates the position of the line-profile analysis; (**b**) Top: NIR-I intensity profiles along the red line. Increasing exposure boosts the ICG signal (peaks) but also elevates the background and induces a false signal in the empty vessel (rightmost region). Bottom: Corresponding LP1000 profiles. ICG peaks remain well separated while baseline and the empty vessel stay at detector noise for all exposures, preserving a high SBR across the full integration range.

The impact of exposure time on ICG signal response was assessed in both the NIR-I and NIR-II channels using the phantom containing a dye-filled branched vessel and a nominally empty control. Four exposures (2 ms, 10 ms, 50 ms, 250 ms) were acquired for each filter set. In the NIR-I channel (Figure 4a), a line profile across three ICG vessels produced high-intensity peaks that broadened and saturated as the integration time increased. Notably, the nominally empty vessel at the bottom of the phantom began to fluoresce at ≥50 ms and was clearly visible at 250 ms. Line-profile analysis (Figure 4b) quantified this behavior, showing that the apparent background rose from 4 × 10^3^ counts at 2 ms to over 7.5 × 10^3^ counts at 250 ms, while the ICG peaks plateaued at 3.2 × 10^4^ counts, leading to a decrease in the SBR from 8.0 to 4.3.

**Figure A3:**
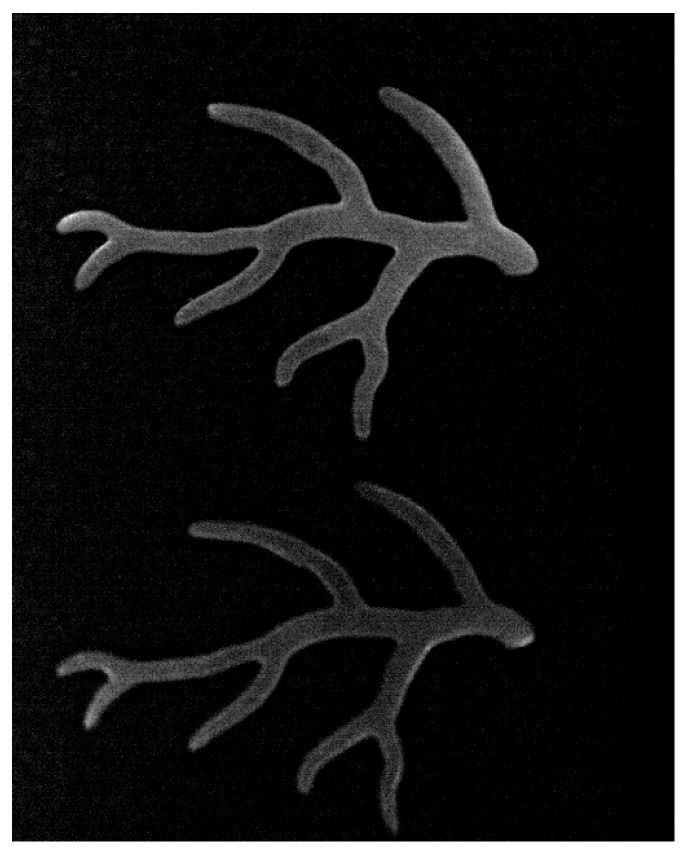
Brightfield reference images of the vessel phantom. Representative brightfield images of a vessel phantom containing ICG-filled channels (top) and an empty channel (bottom).

In contrast, the NIR-II channel (Figure 4a, Appendix A.3, Figure A3) allowed for a clear definition of the ICG vessels across all four exposures, while the empty vessel registered only baseline detector noise. The corresponding line profiles (Figure 4b) indicated that the background never exceeded ~4 × 10^3^ counts, maintaining an SBR above 6 even at the longest integration time. Notably, no spurious fluorescence or scattering artifacts were detectable in the NIR-II dataset.

**Figure 5:**
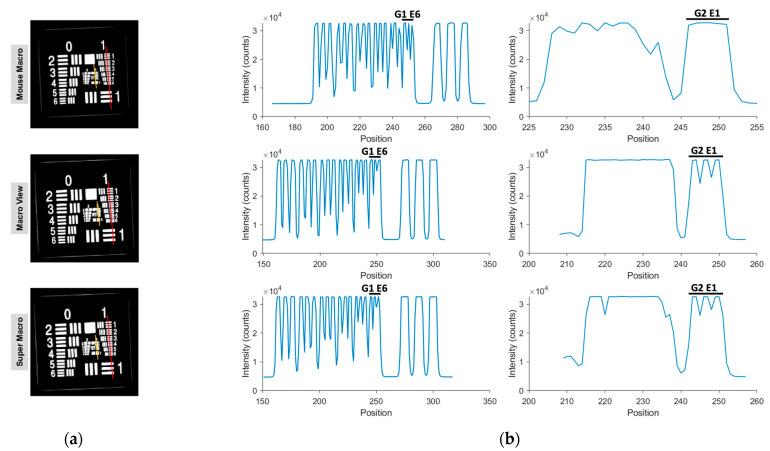
Resolution of the IR-VIVO for ICG. (**a**) 1951 USAF resolution target imaged with the Mouse Macro, Macro View, and Super Macro lenses; (**b**) Line-intensity profiles across the target demonstrating resolution of Group 1 Element 6 (Left, red line ROI) for the Mouse Macro lens and Group 2 Element 1 (Right, Orange line ROI) for both Macro View and Super Macro lenses.

Using a 1951 USAF resolution target, the IR VIVO system resolved Group 1 Element 6 (3.56 lp/mm; ≈140 µm line-pair spacing) with the Mouse Macro lens, whereas both the Macro View and Super Macro lenses resolved Group 2 Element 1 (4.00 lp/mm; 125 µm spacing) (Figure 5a). Line-intensity profiles extracted across the elements (Figure 5b) displayed well-defined high-frequency oscillations that attenuated sharply after the reported elements, confirming the practical resolution limits for each lens configuration.

**Figure 6:**
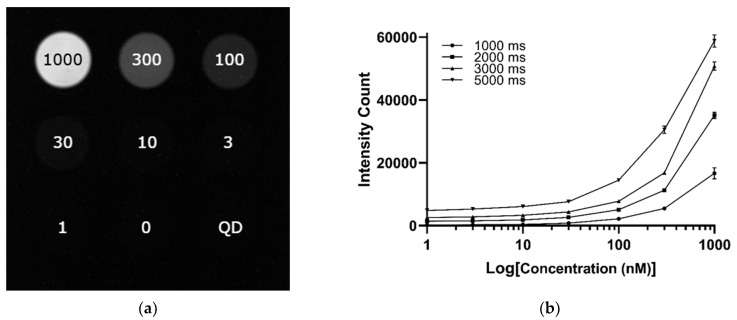

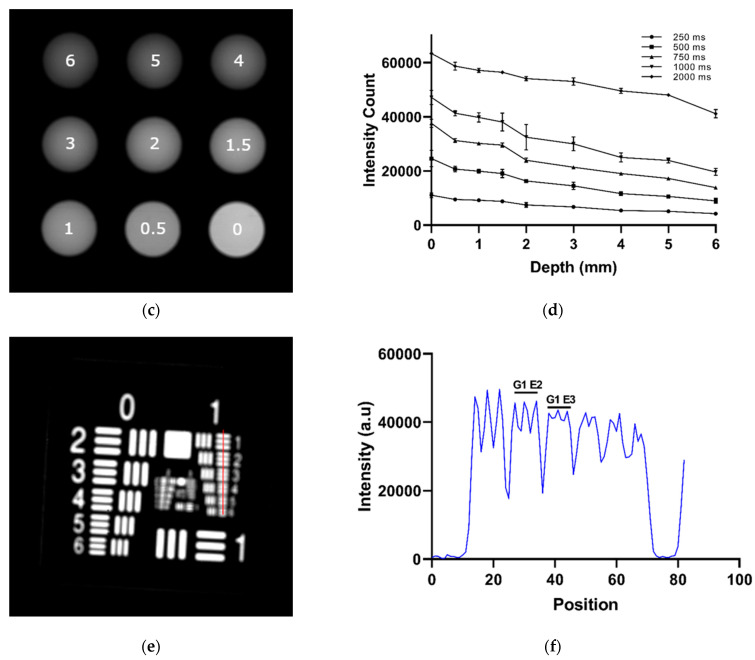
Resolution of the LightIR for ICG. (**a**) QUEL concentration phantom loaded with ICG ranging from 1000 nM to 0 nM, imaged on the LightIR under 1000 ms exposure; (**b**) Quantitative analysis of fluorescence intensity across the concentration gradient of ICG at multiple exposure times, demonstrating a dose-dependent signal response; (**c**) QUEL depth phantom containing 1000 nM ICG, with target depths from 6.0 mm to 0 mm, imaged at 750 ms exposure; (**d**) Plot of fluorescence intensity versus depth under varying exposure times, illustrating signal attenuation with increasing depth; (**e**) QUEL resolution phantom imaged at 850 ms exposure, displaying the spatial resolving capability of the system; (**f**) Quantification of resolution performance across red line in (**e**), showing Group 1 Element 2 as clearly resolved; Group 1 Element 3 appears partially resolved.

To evaluate the imaging performance of the LightIR system in a clinically relevant context, we utilized the QUEL ICG concentration, depth, and resolution phantoms and imaged under standardized conditions. In the concentration phantom (Figure 6a), a serial dilution of ICG from 1000 nM to 0 nM was imaged at a 1000 ms exposure. The resulting image series showed a progressive decrease in fluorescence intensity corresponding to the decreasing ICG concentration. Quantitative analysis across multiple exposure times (Figure 6b) confirmed a concentration-dependent signal response, with higher exposure times yielding improved sensitivity at lower concentrations.

To assess the system’s depth penetration capabilities, the depth phantom filled with 1000 nM ICG was imaged at 750 ms exposure (Figure 6c). Fluorescence signal decreased with increasing depth, as expected. Intensity profiles extracted across different exposure settings (Figure 6d) further illustrated this attenuation, with detectable signal persisting at depths greater than 4 mm.

Resolution performance was evaluated using the standard USAF 1951 resolution target imaged at 850 ms exposure time (Figure 6e). The imaging system clearly resolved Group 1, Element 2 (2.24 lp/mm; 223 µm). Partial resolution of Group 1, Element 3 (2.52 lp/mm; 198 µm) was also observed. Line profile analysis across the resolution pattern (Figure 6f) demonstrated well-defined intensity peaks and troughs for Element 2, with reduced contrast between bars in Element 3, indicating the system’s resolution threshold approaches 198 µm under the given conditions.

**Figure 7:**
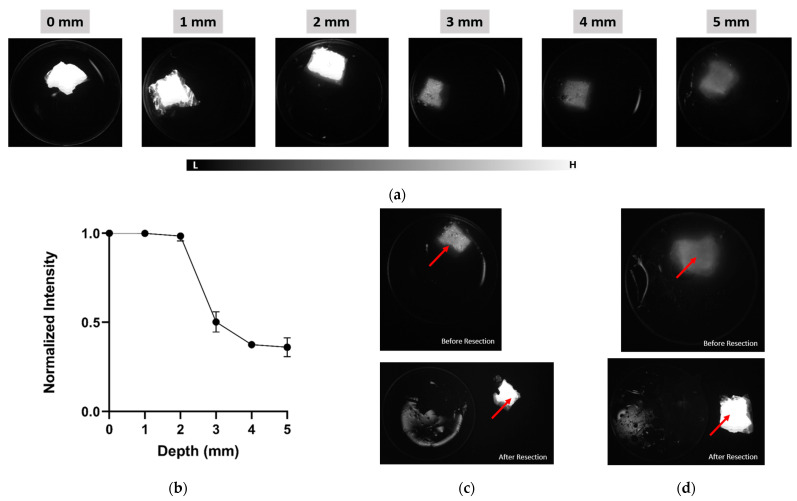
Depth sensitivity of LightIR for IR-1048 in the NIR-II window. (**a**) NIR-II fluorescence images of IR-1048-soaked sponge embedded at depths ranging from 0 mm to 5 mm in agar-based tissue-mimicking phantoms (1% Intralipid, 0.01% India ink) Red arrow indicates location of the tumor mimicking sponge. (**b**) Quantitative analysis of normalized fluorescence intensity as a function of depth, illustrating signal attenuation due to scattering and absorption. (**c**) Still frame from real-time fluorescence-guided surgery (FGS) under ambient light conditions showing IR-1048 resection at 3 mm depth. (**d**) Corresponding FGS at 5 mm depth, with a weaker but detectable fluorescence signal guiding successful excision. Imaging was performed using the LightIR system with a 1050 nm long-pass filter and pulse-mode background subtraction under ambient lighting.

To evaluate depth-resolved imaging capability using IR-1048, agar-based phantoms containing 1% Intralipid and 0.01% India ink were prepared with embedded dye samples positioned at depths ranging from 0 mm to 5 mm. Figure 7a displays representative fluorescence images acquired at each depth with a 500-ms exposure time. IR-1048 remained clearly visible up to 3 mm, with gradually diminishing intensity at 4 mm and a faint but detectable signal at 5 mm. Quantitative analysis (Figure 7b) confirmed a monotonic decline in normalized fluorescence intensity with increasing depth, reflecting expected photon attenuation in turbid media. Real-time fluorescence-guided surgery (FGS) was then performed on embedded dye samples at 3 mm and 5 mm depths (Figure 7c, Video S1 and Figure 7d, Video S2, respectively). At 3 mm, the dye was sharply visualized and cleanly resected under NIR-II guidance. At 5 mm, the fluorescence signal was weaker, but the dye could still be localized and surgically excised based on NIR-II contrast.

Performance comparison between the IR VIVO and LightIR systems using standardized ICG phantoms is summarized in Table 1. In terms of depth sensitivity, IR VIVO imaging with LP1000 at 808 nm maintained stable signal-to-background ratios (SBRs) of 2.0–2.5 from 0 to 6 mm depth, while NIR-I detection showed a higher initial SBR (6.5 at 0 mm) that declined below 2.0 beyond 4 mm. LightIR, operating under ambient light with an integrated LP1050 filter, achieved comparable depth sensitivity, detecting ICG to ≥4 mm at 750 ms exposure. For concentration sensitivity, IR VIVO detected ICG down to 30 nM in NIR-I and 300 nM in NIR-II (200 ms), while LightIR achieved a detection threshold of 100 nM in NIR-II (1000 ms). Spatial resolution was higher on IR VIVO, which resolved Group 2, Element 1 (4.00 lp/mm; 125 µm) at 50 ms, compared to LightIR’s resolution of Group 1, Element 2 (1.78 lp/mm; 281 µm) with partial resolution of Element 3 (250 µm) at 850 ms.

## 4. Discussion

In this study, we investigated the sensitivity of near-infrared (NIR) imaging utilizing the IR VIVO system across both the NIR-I and NIR-II spectral windows. To achieve this, we employed a nine-well QUEL concentration phantom, containing varying concentrations of indocyanine green (ICG) ranging from 0 to 1000 nM, alongside a quantum-dot (QD) positive control. The QUEL phantoms are designed to mimic ICG fluorescence in the presence of tissue optical properties. We did not observe much difference when ICG was excited by either a 760 nm or 808 nm laser and imaged using an NIR-I filter. When excited with the same lasers but imaged using a long-pass 1000 nm filter, the signal-to-background (SBR) ratio decreased relative to ICG excited by the 808 or 750 nm laser. This is as expected, as ICG has a maximum absorption at 808 nm. However, Figure 2 shows that for the same concentrations, the SBR is higher in the NIR-I window than in the NIR-II for ICG. A similar trend is observed when we compare the QUEL depth phantoms in Figure 3; the SBR is higher when imaged in NIR-I as compared to NIR-II with the same exposure time. However, a more comparable result is to adjust the initial settings such that we get the same amount of initial photon influx. When excited with the 808 nm laser but using an exposure time of 25 ms for the NIR-I window and 200 ms for the LP1000 (NIR-II) window, we have an SBR of 5.5 in both channels for 1000 nM of ICG at 0 mm depth. Under these conditions, the signal of ICG in the NIR-I window plateaus with an SBR of 2.1 after 5 mm, while in the NIR-II window, we still get an SBR of 3.5 at 6 mm depth of tissue mimicking phantoms. These results show that with just a tail emission in the NIR-II region, the current FDA-approved ICG might not be the most viable candidate for NIR-II imaging, where only a small percentage of the photons can be imaged in that window, requiring a much higher exposure time. It is important to note that our results are limited to just phantom studies in this paper; ICG in the presence of proteins can have different behavior. Imaging agents with the maximum absorption in the NIR-II window will be required in order to harness the benefits of imaging in the NIR-II window. When comparing ICG and IR-1048 in phantom studies, we observed that the limit of detection for ICG in the NIR-II window appeared to plateau around 4 mm, whereas IR-1048 maintained a detectable signal even at depths of 5 mm (Figure 6 and Figure 7). This highlights a critical distinction between a dye like ICG that only exhibits weak tail emission beyond 1000 nm and IR-1048, with its peak absorption and emission fully within the NIR-II window. These results underscore that imaging depth is not solely dictated by the capabilities of the camera system but also fundamentally influenced by the photophysical properties of the dye itself. Therefore, fluorophores with strong NIR-II emission profiles and higher quantum yields are expected to outperform dyes like ICG in applications requiring deeper tissue visualization.

Both the IR VIVO and LightIR systems utilize the Alizé 1.7 camera, enabling sensitivity in the NIR-II window; however, their form factors and operational designs differ significantly. The IR VIVO has been more thoroughly validated in the literature, with numerous publications demonstrating its performance in in VIVO tumor and vasculature imaging [22,25,30,48,49,50,51,52,53,54,55]. Its integrated imaging chamber, which fully shields the sample from ambient light, ensures high signal-to-background ratios (SBR) and reproducibility under controlled conditions. In contrast, the LightIR is a newer, more compact system designed with clinical translation in mind, and we sought to evaluate whether it could retain comparable imaging performance across a series of standardized NIR-II phantoms.

To assess this, we performed a head-to-head comparison of both systems using QUEL ICG concentration and depth penetration phantoms, and a USAF 1951 resolution target. Notably, when imaging the concentration phantom, the LightIR required longer acquisition times to achieve comparable signal intensity and dynamic range, likely due to the lack of full ambient light exclusion (Figure 2 and Figure 6a,b). Similarly, for depth phantoms, the IR VIVO consistently provided higher contrast for the same depths, whereas the LightIR showed decreased sensitivity unless longer integration and exposure times were employed (Figure 3 and Figure 6c,d). In resolution phantoms, subtle differences in edge definition and sharpness were observed, with the IR VIVO offering slightly better resolution under default settings (Figure 5 and Figure 6e,f). These differences emphasize the role of environmental control in optimizing image quality, particularly in low-signal NIR-II applications.

An important distinction between the two systems lies in the imaging setup and environmental lighting. The IR VIVO system is fully enclosed, operating in complete darkness to eliminate background light contamination. This provides a stable and highly controlled imaging environment ideal for sensitive fluorescence measurements. In contrast, the LightIR was operated in an open environment, under standard ceiling room lights, and used its built-in “pulse mode” to acquire alternating brightfield and fluorescence images. The system then generated subtracted composite images, enhancing contrast by removing ambient signal contributions. While this mode is advantageous in settings where darkroom conditions are impractical, such as clinical operating rooms, it introduces variability and may necessitate higher dye concentrations or longer acquisition times to compensate for ambient light interference.

While both the IR VIVO and LightIR systems offer control over key imaging parameters, such as camera exposure, gain, laser type, and intensity, the LightIR was specifically engineered with clinical usability in mind, resulting in a more streamlined and intuitive user interface. Both platforms support programmable acquisition sequences to automate imaging workflows; however, the LightIR software - KIS control (v3.0) presents simplified menus, real-time image processing, and easily accessible imaging modes, reducing the learning curve for surgical teams. Unlike the IR VIVO, which requires software-based control of camera position and filter selection, LightIR allows for quick manual adjustments of camera height and filter changes, an advantage in fast-paced intraoperative environments. These design choices reflect the system’s focus on ease-of-use and rapid deployment, making it more adaptable for back table and real-time surgical imaging compared to its preclinical counterpart. This user-centered approach positions LightIR as a practical solution for fluorescence-guided procedures in the operating room.

Overall, while both systems leverage the same core detector technology, the IR VIVO’s optimized enclosure and software presets offer clear advantages for preclinical, high-sensitivity imaging. However, the LightIR’s portability and capacity to perform in ambient light settings, albeit with some trade-offs in sensitivity and temporal resolution, make it a promising platform for translation to clinical environments. Our comparative results support the use of LightIR in surgical or bedside contexts, especially when combined with appropriate imaging protocols to mitigate the impact of ambient light.

## 5. Conclusions

The IR VIVO system has now been in circulation for years for preclinical NIR-II fluorescence imaging, with numerous publications validating its use in small-animal tumor and vasculature imaging. In this study, we aimed to assess whether comparable performance could be achieved using the LightIR system, a more compact and clinically oriented imaging platform that shares the same Alizé 1.7 InGaAs detector. Through standardized phantom studies, we demonstrated that LightIR retains many of the imaging strengths of the IR VIVO, including submillimeter resolution and depth sensitivity up to 5 mm, even under ambient light using pulse-mode acquisition. While the IR VIVO remains advantageous in controlled laboratory environments with shorter exposure times and higher spatial fidelity, the LightIR enables real-time fluorescence imaging in open surgical fields, presenting a practical and translational solution for intraoperative NIR-II applications. By validating LightIR’s performance in side-by-side comparisons, this work supports its use not only in clinical workflows but also as a versatile testbed for future probe evaluation.

Despite the growing interest in NIR-II imaging, the availability of suitable fluorophores has lagged behind, in part due to the lack of robust and accessible imaging systems optimized for this spectral window. Historically, the limited adoption of NIR-II platforms may have discouraged the widespread development of dyes that absorb and emit entirely beyond 1000 nm. With clinically compatible systems like the LightIR now available, there is a strong incentive to advance the chemistry of NIR-II fluorophores to match the capabilities of modern detectors and filters. While ICG was used here for baseline comparisons, its weak tail emission in the NIR-II region limits both depth penetration and signal-to-background ratios. Moving forward, the development of dedicated NIR-II probes with peak absorption and emission in the 1000–1350 nm range will be critical to fully realize the well-documented benefits of this imaging window—including enhanced tissue penetration, lower autofluorescence, and improved spatial resolution. The convergence of better instrumentation and targeted probe development offers an exciting opportunity to accelerate the clinical translation of NIR-II fluorescence-guided surgery.

This study establishes a translational pipeline connecting NIR-II fluorophore development to intraoperative imaging, demonstrating that the LightIR can match the IR VIVO performance while operating under ambient light. Its compact design, real-time background subtraction, and submillimeter resolution make it a promising tool for back table imaging during surgery. Moving forward, we aim to validate the LightIR in large animal models with tumor xenografts and freshly resected human specimens. Future iterations of the system will incorporate a surgical arm, enabling real-time cavity or patient imaging and expanding its role beyond specimen analysis to full intraoperative guidance.

## Figures and Tables

**Table 1:** Comparative performance of the IR VIVO and LightIR imaging systems using standardized ICG phantoms. The table summarizes key imaging metrics, including depth sensitivity, concentration sensitivity, and spatial resolution. IR VIVO demonstrated higher spatial resolution and lower detection limits under controlled conditions, with better performance at short exposure times. LightIR, designed for ambient light intraoperative use, achieved comparable depth sensitivity but required longer exposure times due to its integrated LP1050 filter, which blocks most of ICG’s NIR-I emission.

Parameter	IR VIVO	LightIR ^1^
Depth Sensitivity	808 nm LP1000 (NIR-II) maintained SBRs of 2.0–2.5 from 0 to 6 mm with sharper boundaries, while 808 nm NIR-I showed higher initial SBR (6.5 at 0 mm) but dropped below 2.0 beyond 4 mm with increased blurring (Exposure time: 200 ms)	Detectable ICG signal to 4 mm or more with LP1050 at 808 nm excitation (Exposure time: 750 ms)
Concentration Sensitivity	Detectable ICG down to approximately 30 nM in NIR-I; limit of detection in NIR-II approximately 300 nM (Exposure time: 200 ms)	Detectable ICG down to 100 nM in NIR-II (Exposure time: 1000 ms)
Spatial Resolution	Resolved Group 2, Element 1 (4.00 lp/mm; approximately 125 μm) with Macro/Super Macro lens (Exposure time: 50 ms)	Resolved Group 1, Element 2 (1.78 lp/mm; approximately 281 μm); partial resolution of Element 3 (approximately 250 μm) (Exposure time: 850 ms)

^1^ Used under ambient light conditions.

## Data Availability

Data supporting the present study are available from the corresponding author upon reasonable request.

## Supplementary Materials

The following supporting information can be downloaded at https://www.mdpi.com/article/10.3390/cancers17162676/s1: Video S1: Simulated NIR-II Fluorescence-Guided Tumor Resection at 3 mm Depth (Phantom Model), Video S2: Simulated NIR-II Fluorescence-Guided Tumor Resection at 5 mm Depth (Phantom Model).

## Abbreviations

The following abbreviations are used in this manuscript:

NIR	Near-Infrared
NIR-I	First Near-Infrared Window (700–950 nm)
NIR-II	Second Near-Infrared Window (1000–1700 nm)
FGS	Fluorescence-Guided Surgery
ICG	Indocyanine Green
SBR	Signal-to-Background Ratio
ROI	Region of Interest
LP	Long-Pass
InGaAs	Indium Gallium Arsenide

## Appendix A

### Appendix A.4

**Table A1:** Power density distribution across stage positions for distinct laser sources.

		Power Density in mW/mm^2^			
	Excitation Laser	760 nm	808 nm	890 nm	940 nm
Stage Position					
3 mice		1.00	1.10	0.84	1.15
2 mice		1.63	1.80	1.59	2.14
1 mouse wide		2.00	2.33	1.94	2.66
1 mouse macro		1.35	1.44	1.18	1.65
Macro View		1.93	2.26	1.94	2.64
Super Macro View		2.02	2.37	2.02	2.80
